# Supplementary material for: Antioxidant, Neuroprotective, and Antinociceptive Effects of Peruvian Black Maca (Lepidium meyenii Walp.)
Source: Antioxidants (Basel). 2025 Oct 8;14(10):1214. doi: 10.3390/antiox14101214 (PMC12561524; doi:10.3390/antiox14101214)
Supplement: Supplementary file 1 [file antioxidants-14-01214-s001.zip › antioxidants-3888947-supplementary.pdf]

Antioxidant, Neuroprotective, and Antinociceptive Effects of Peruvian Black Maca (*Lepidium meyenii* Walp.)

Iván M. Quispe-Díaz [1], Roberto O. Ybañez-Julca [1,*], Daniel Asunción-Alvarez [1], Cinthya Enriquez-Lara [2], José L. Polo-Bardales [3], Rafael Jara-Aguilar [3], Edmundo A. Venegas-Casanova [3], Ricardo D.D.G. de Albuquerque [4], Noé Costilla-Sánchez [5], Edison Vásquez-Corales [6], Pedro Buc Calderon [2,7] and Julio Benites [2,8*]

1 Grupo de Investigación en Estudios de Compuestos Naturales y Sintéticos con Actividad a Nivel Sistema Nervioso Central y Musculo Liso, Laboratorio de Farmacología, Facultad de Farmacia y Bioquímica, Universidad Nacional de Trujillo, Trujillo 13011, Peru; iquispe@unitru.edu.pe (I.M.Q.-D.); hasuncion@unitru.edu.pe (D.A.-A.)
2 Programa de Doctorado en Química Medicinal, Facultad de Ciencias de la Salud, Universidad Arturo Prat, Casilla 121, Iquique 1110939, Chile; cenriquez@estudiantesunap.cl (C.E.-L.); pedro.buccalderon@uclouvain.be (P.B.C.)
3 Facultad de Farmacia y Bioquímica, Universidad Nacional de Trujillo, Trujillo 13011, Peru; jpolo@unitru.edu.pe (J.L.P.-B.); djara@unitru.edu.pe (R.J.-A.); evenegas@unitru.edu.pe (E.A.V.-C.)
4 Laboratório de Tecnologia em Produtos Naturais, Universidade Federal Fluminense, Niterói 24020-140, RJ, Brazil; richardcabofrio@gmail.com
5 Laboratorio de Métodos Instrumentales, Facultad de Ingeniería Química, Universidad Nacional de Trujillo, Trujillo 13011, Peru; ncostilla@unitru.edu.pe
6 Escuela de Farmacia y Bioquímica, Universidad Católica Los Ángeles de Chimbote, Chimbote 02801, Peru; evasquezc@uladech.edu.pe
7 Research Group in Metabolism and Nutrition, Louvain Drug Research Institute, Université Catholique de Louvain, 73 Avenue E. Mounier, GTOX 7309, 1200 Brussels, Belgium
8 Laboratorio de Química Medicinal, Química y Farmacia, Facultad de Ciencias de la Salud, Universidad Arturo Prat, Casilla 121, Iquique 1110939, Chile
* Correspondence: rybanez@unitru.edu.pe (R.O.Y.-J.); juliob@unap.cl (J.B.); Tel.: +51-0449-7634-5993 (R.O.Y.-J.); +56-57-2252-6275 (J.B.)

SUPPORTING INFORMATION

Figure S1. **.** UHPLC-ESI-Q-TOF-MS total ion chromatograms of Black Maca*: Lepidium meyenii*.
A) positive ionization mode and **B**) negative ionization mode
Metabolite profiling of by UHPLC-ESI-Q-TOF-MS.

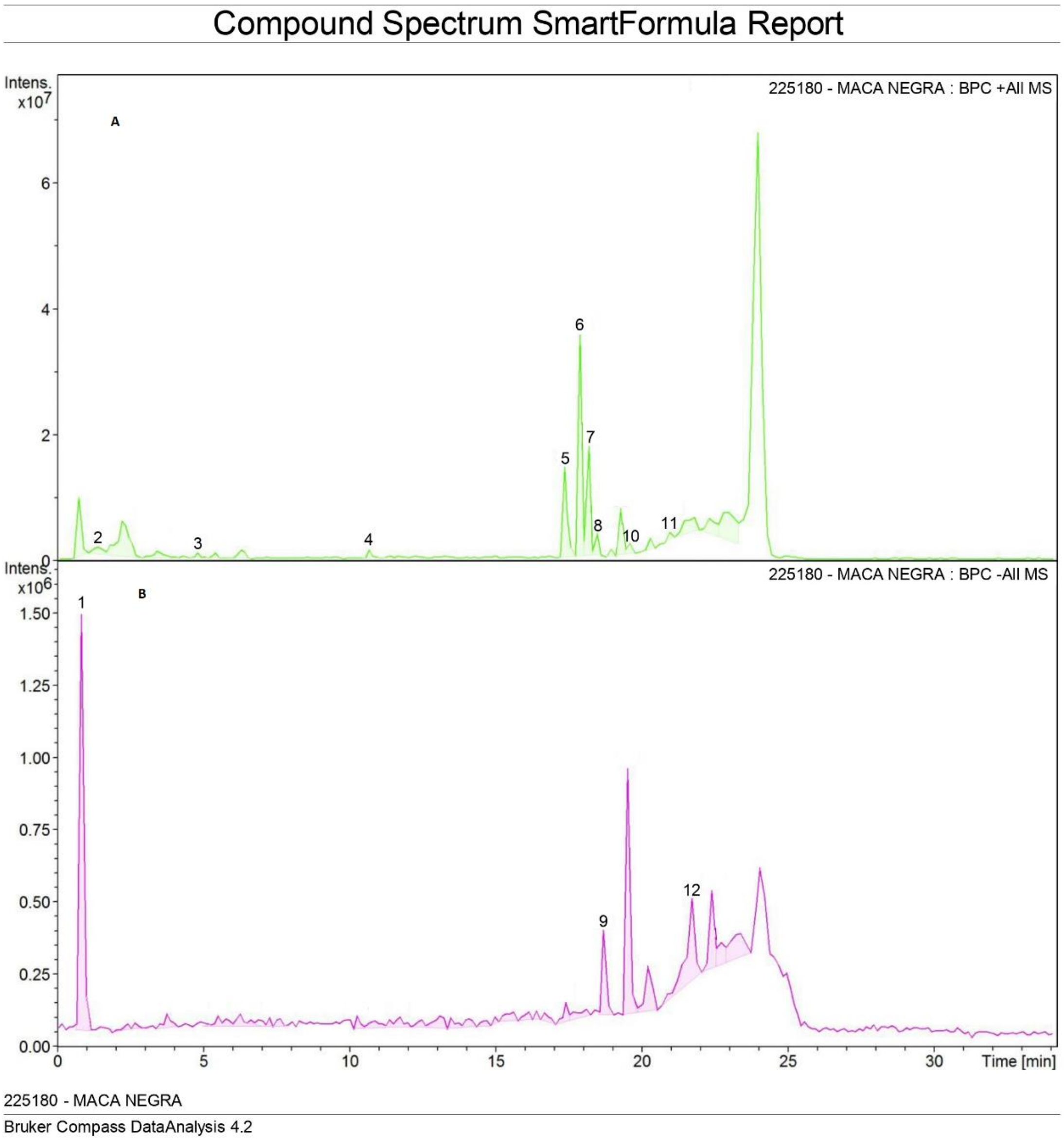

Figure S2. 2D representation of the interactions between compounds **1-12** and residues of NADPH oxidase (PDB ID: 2CDU). Hydrogen atoms have been omitted in some cases for clarity.

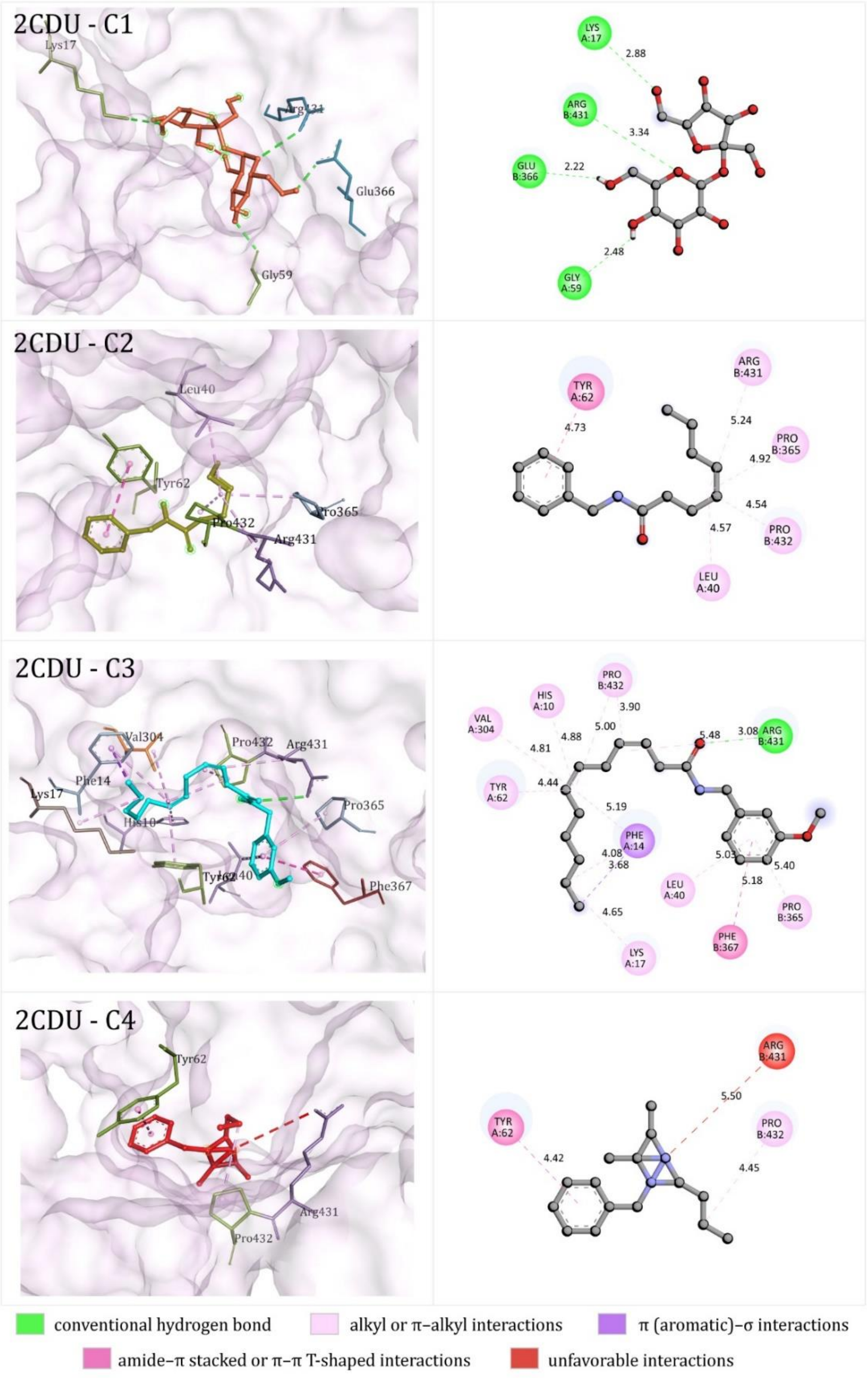

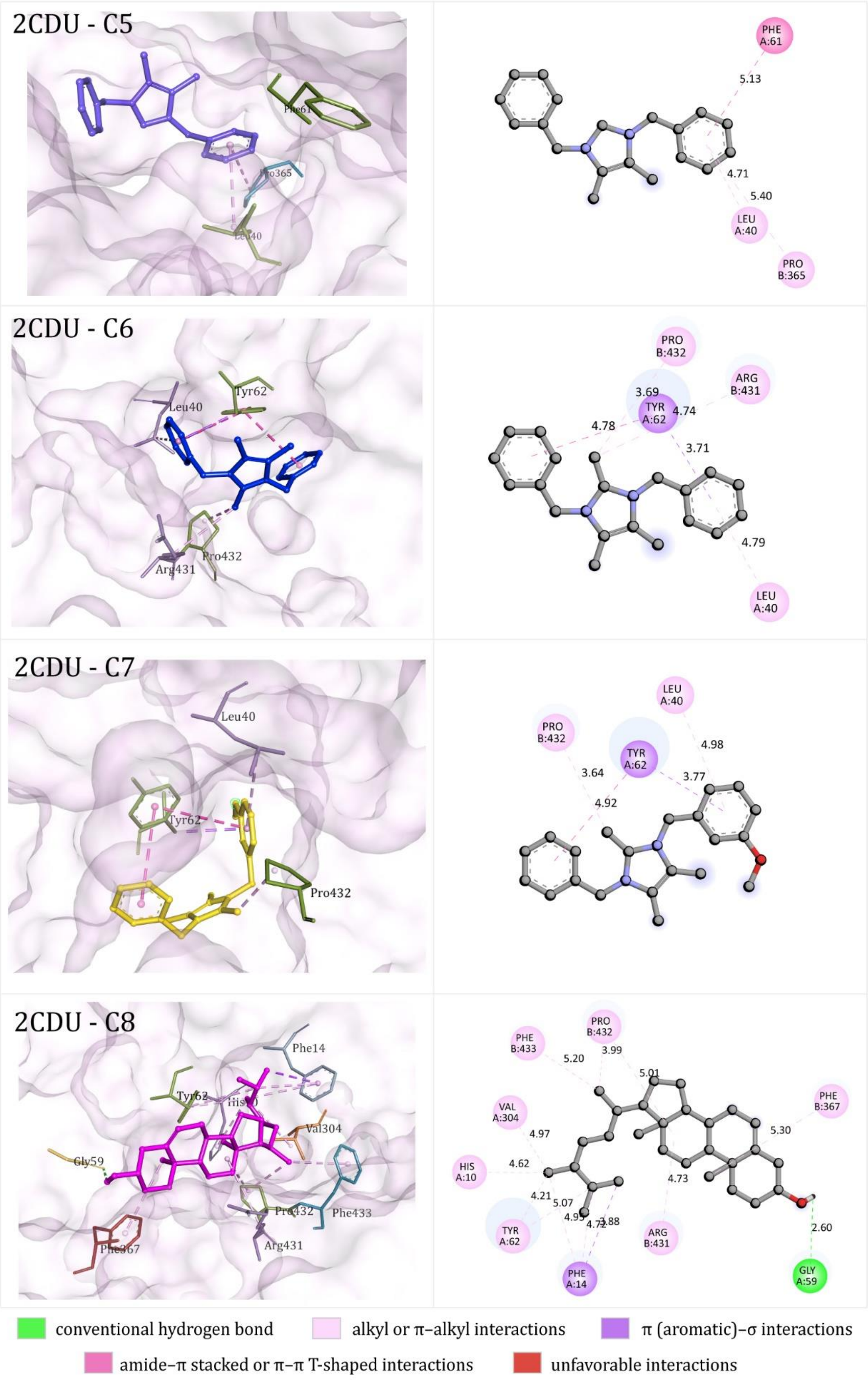
2CDU - C5
Phe61
Pro365
Leu40
PHE A:61
5.13
4.71
5.40
LEU A:40
PRO B:365
2CDU - C6
Tyr62
Leu40
Pro432
Arg431
PRO B:432
ARG B:431
3.69
TYR A:62
4.74
4.78
3.71
4.79
LEU A:40
2CDU - C7
Leu40
Tyr62
Pro432
LEU A:40
PRO B:432
TYR A:62
4.98
3.64
3.77
4.92
2CDU - C8
Phe14
Tyr62
His10
Val304
Gly59
Pro432
Phe433
Phe367
Arg431
PRO B:432
PHE B:433
5.20
3.99
5.01
VAL A:304
4.97
PHE B:367
5.30
HIS A:10
4.62
4.73
4.21
5.07
4.95
4.72
3.88
TYR A:62
ARG B:431
2.60
PHE A:14
GLY A:59
conventional hydrogen bond
alkyl or π–alkyl interactions
π (aromatic)–σ interactions
amide–π stacked or π–π T-shaped interactions
unfavorable interactions

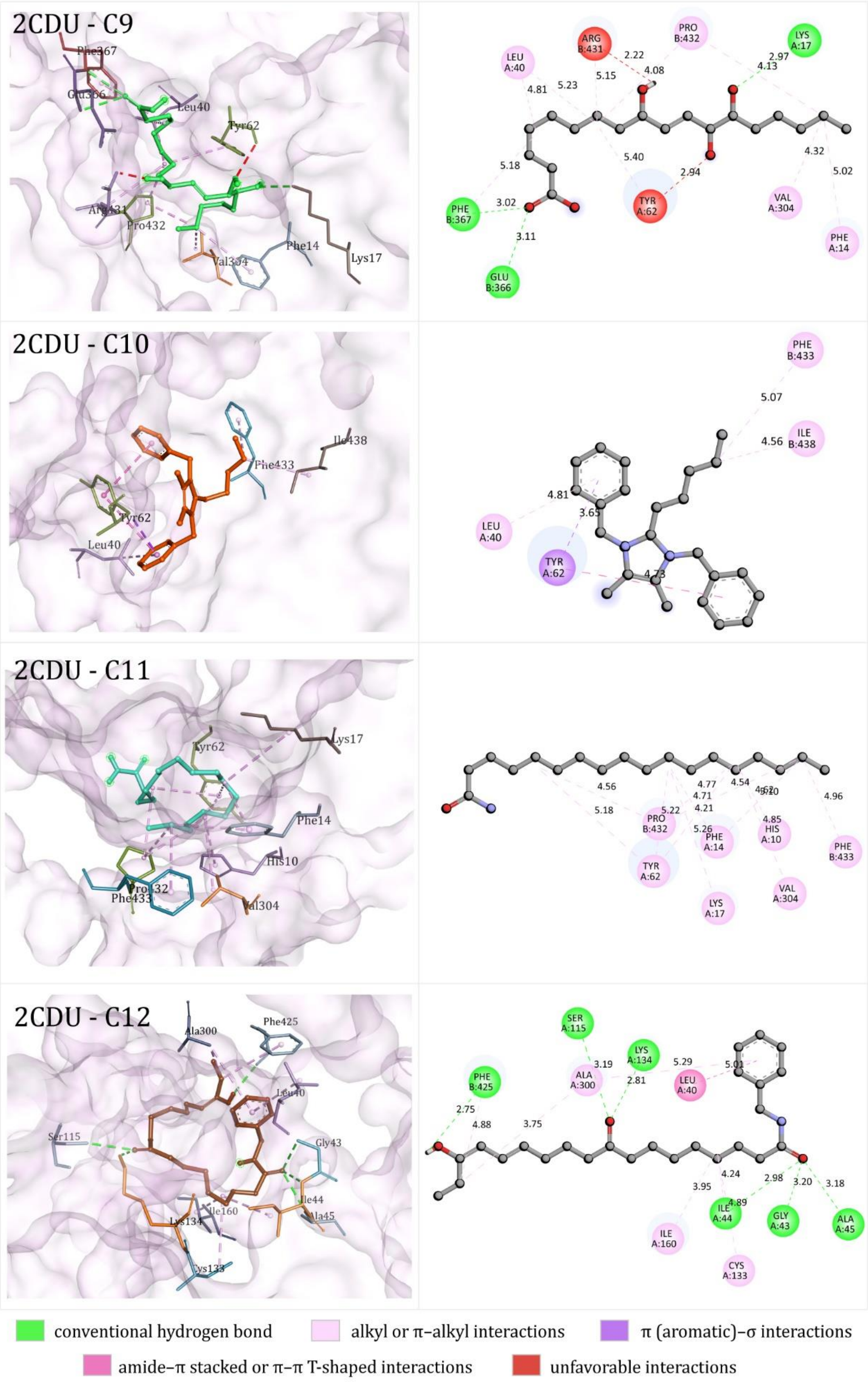
2CDU - C9
2CDU - C10
2CDU - C11
2CDU - C12
conventional hydrogen bond
alkyl or π–alkyl interactions
π (aromatic)–σ interactions
amide–π stacked or π–π T-shaped interactions
unfavorable interactions

Figure S3. 2D representation of the interactions between compounds 1-12 and residues of xanthine oxidase (PDB ID: 3NRZ). Hydrogen atoms have been omitted in some cases for clarity.

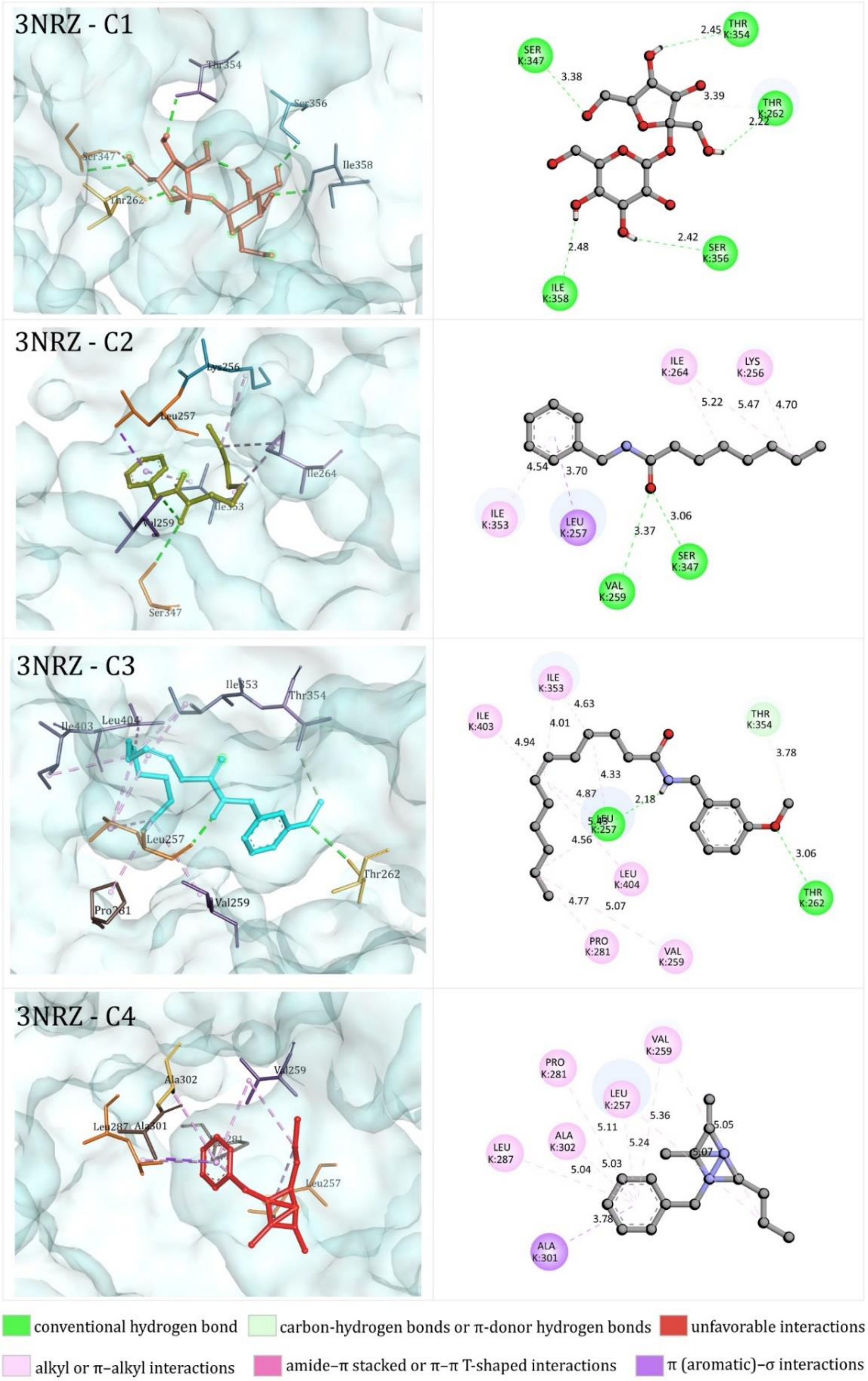

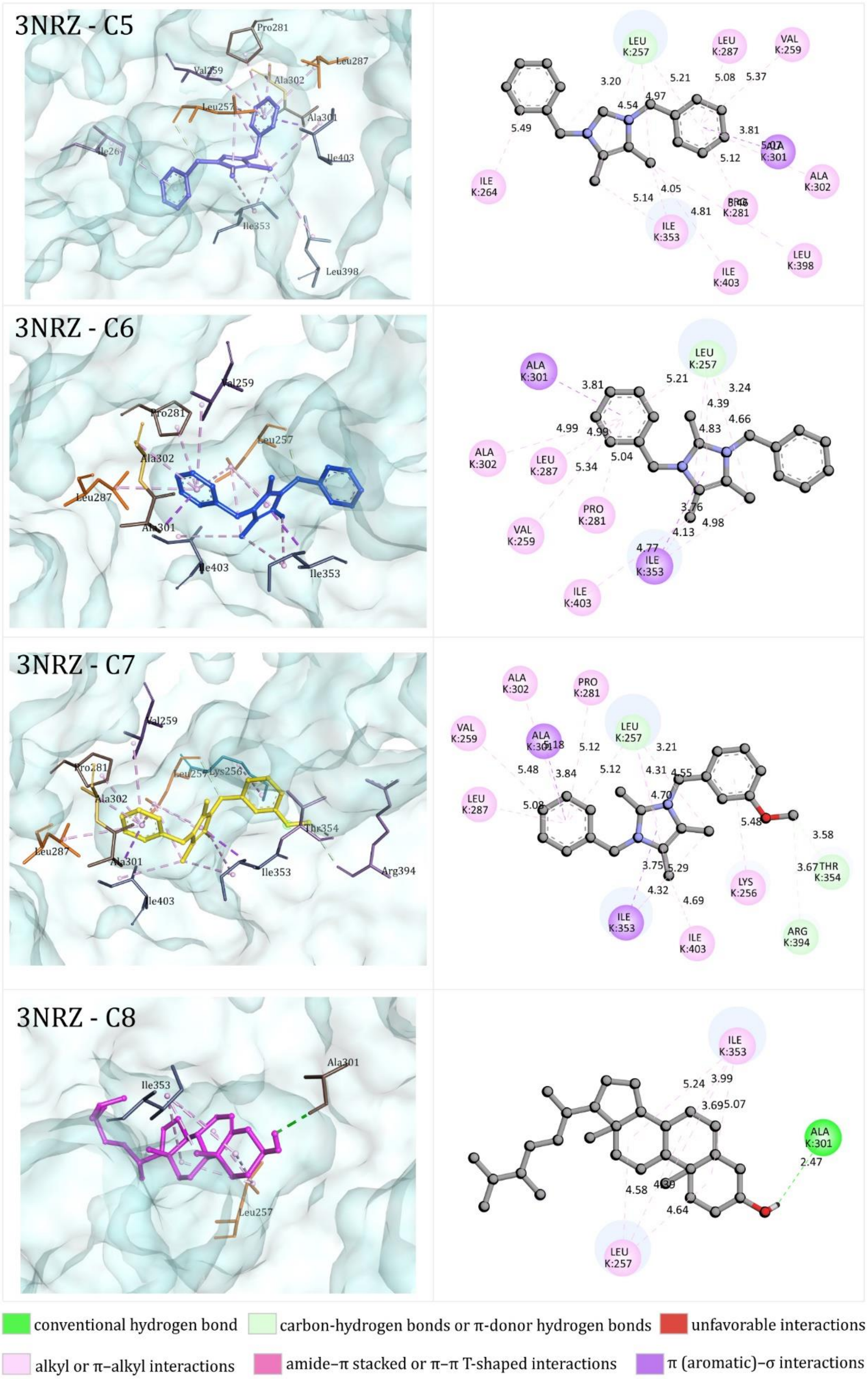
3NRZ - C5
3NRZ - C6
3NRZ - C7
3NRZ - C8
conventional hydrogen bond
carbon-hydrogen bonds or π-donor hydrogen bonds
unfavorable interactions
alkyl or π–alkyl interactions
amide–π stacked or π–π T-shaped interactions
π (aromatic)–σ interactions

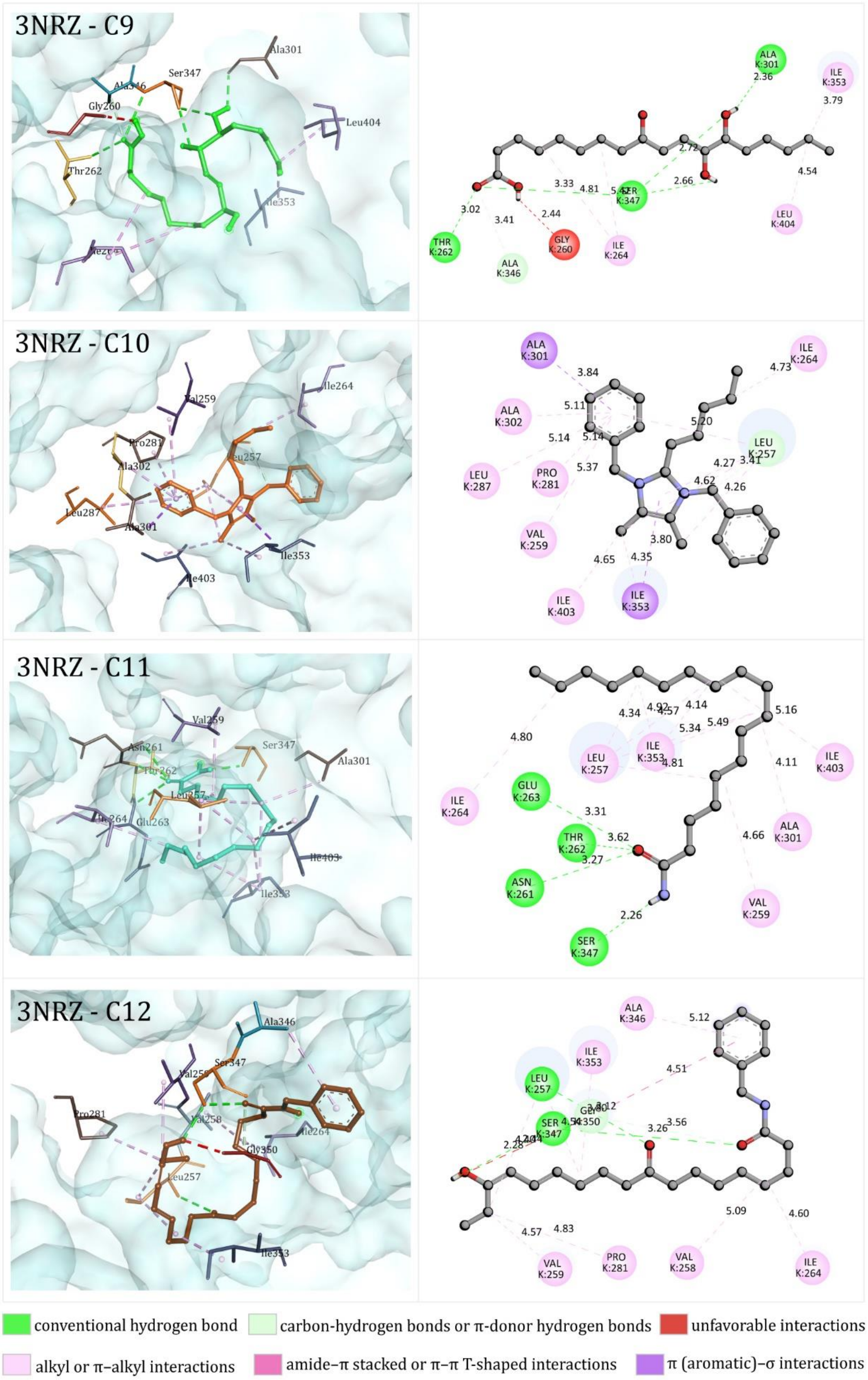
3NRZ - C9
3NRZ - C10
3NRZ - C11
3NRZ - C12
conventional hydrogen bond
carbon-hydrogen bonds or π-donor hydrogen bonds
unfavorable interactions
alkyl or π–alkyl interactions
amide–π stacked or π–π T-shaped interactions
π (aromatic)–σ interactions

Figure S4. 2D representation of the interactions between compounds **1-12** and residues of superoxide dismutase (PDB ID: 4MCM). Hydrogen atoms have been omitted in some cases for clarity.

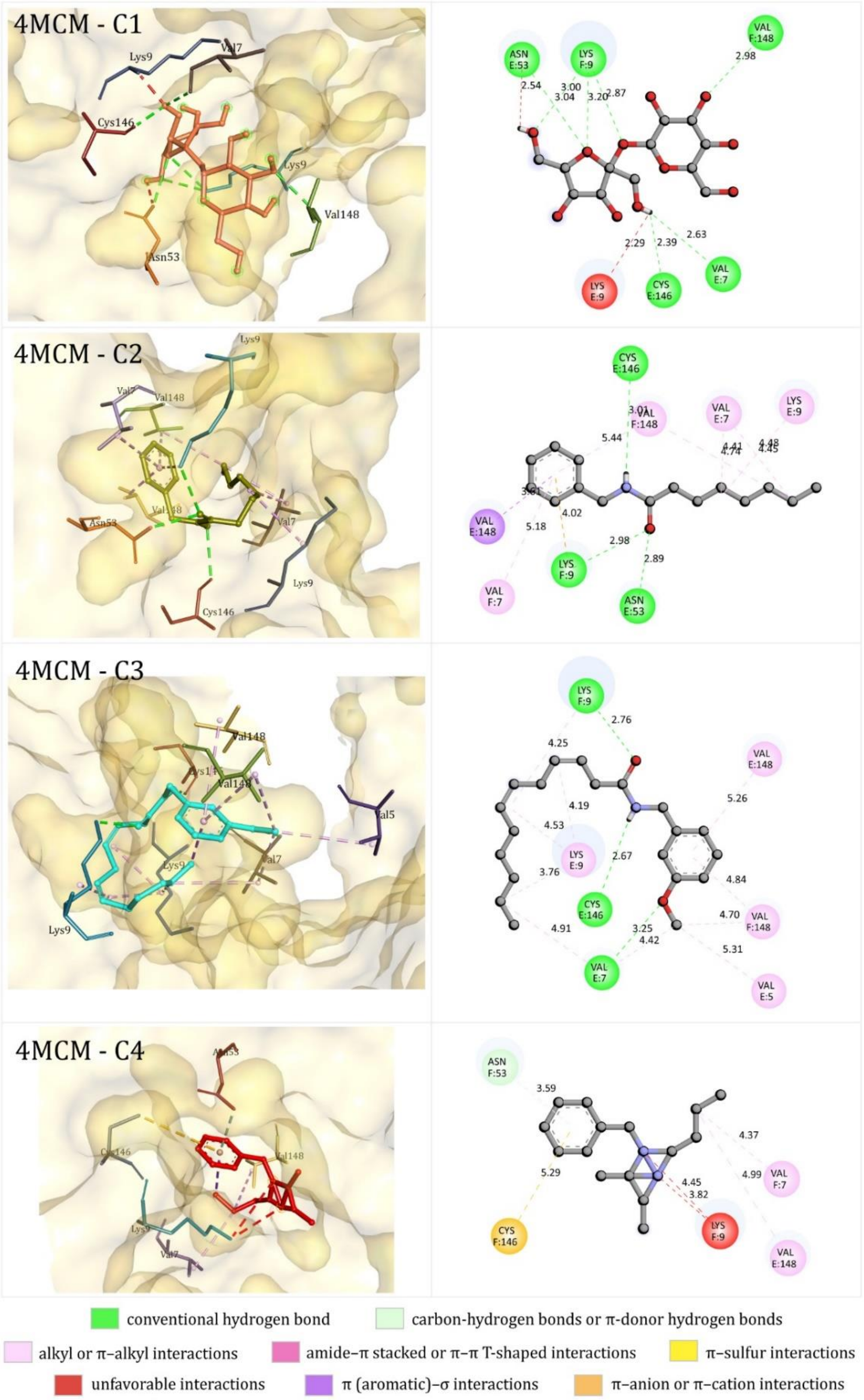

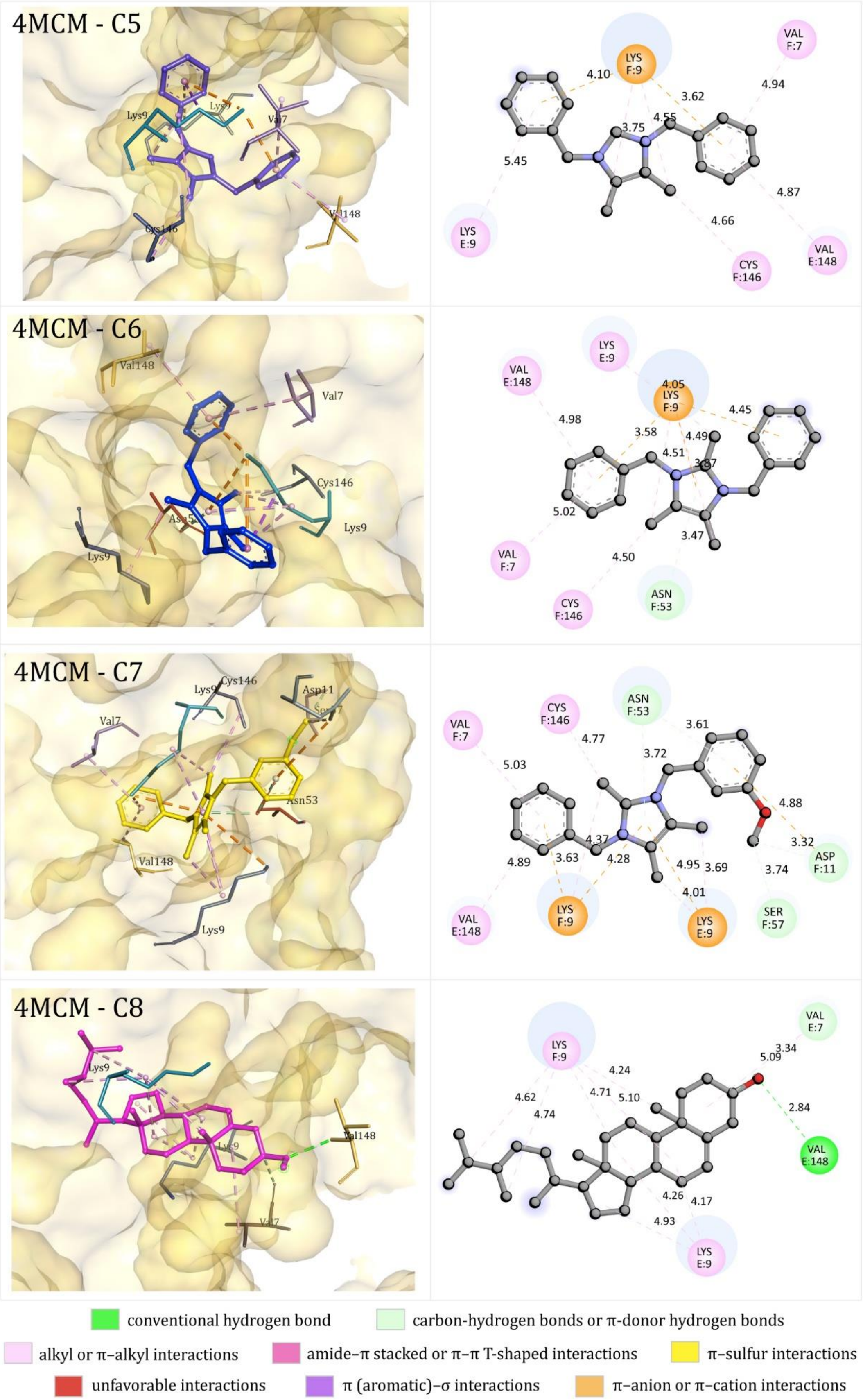

4MCM - C5
4MCM - C6
4MCM - C7
4MCM - C8
conventional hydrogen bond
carbon-hydrogen bonds or π-donor hydrogen bonds
alkyl or π–alkyl interactions
amide–π stacked or π–π T-shaped interactions
π–sulfur interactions
unfavorable interactions
π (aromatic)–σ interactions
π–anion or π–cation interactions

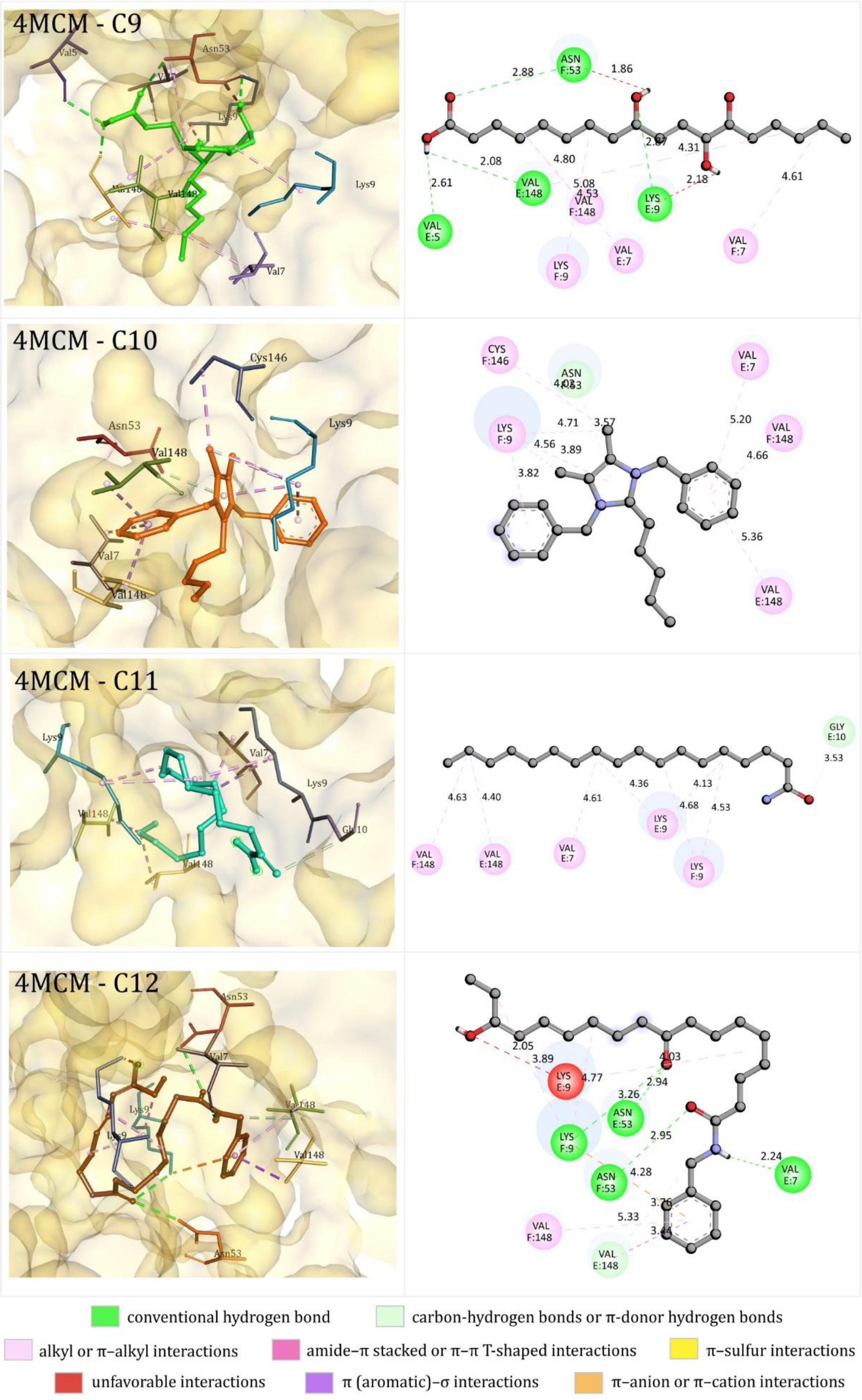
4MCM - C9
4MCM - C10
4MCM - C11
4MCM - C12
ASN F:53
VAL E:148
VAL E:5
VAL F:148
LYS E:9
VAL E:7
LYS F:9
VAL F:7
CYS F:146
VAL F:148
GLY E:10
ASN E:53
ASN F:53
conventional hydrogen bond
carbon-hydrogen bonds or π-donor hydrogen bonds
alkyl or π–alkyl interactions
amide–π stacked or π–π T-shaped interactions
π–sulfur interactions
unfavorable interactions
π (aromatic)–σ interactions
π–anion or π–cation interactions

Figure S5. 2D representation of the interactions between compounds **1-12** and residues of the μ-opioid receptor (PDB ID: 4DKL). Hydrogen atoms have been omitted in some cases for clarity.

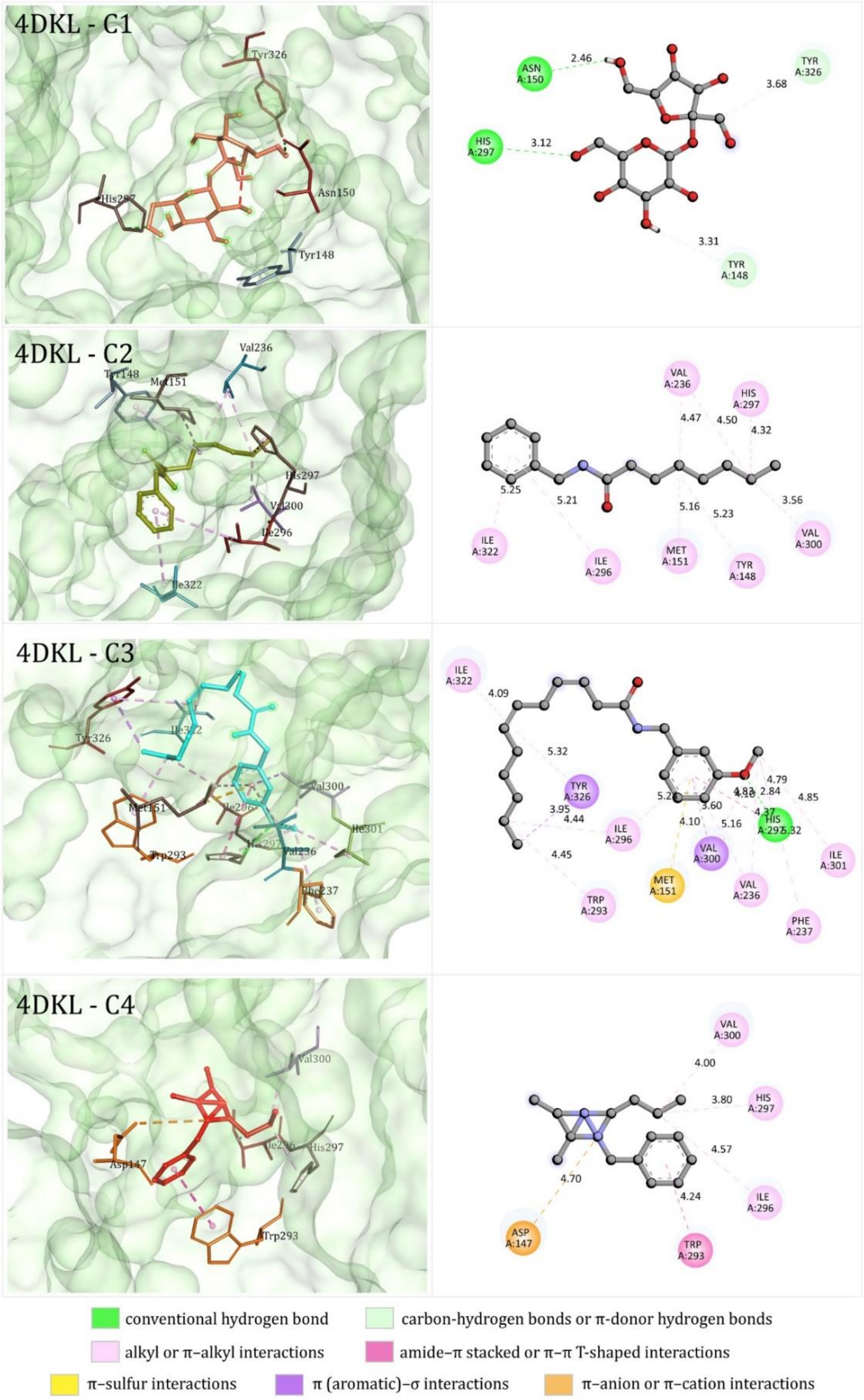

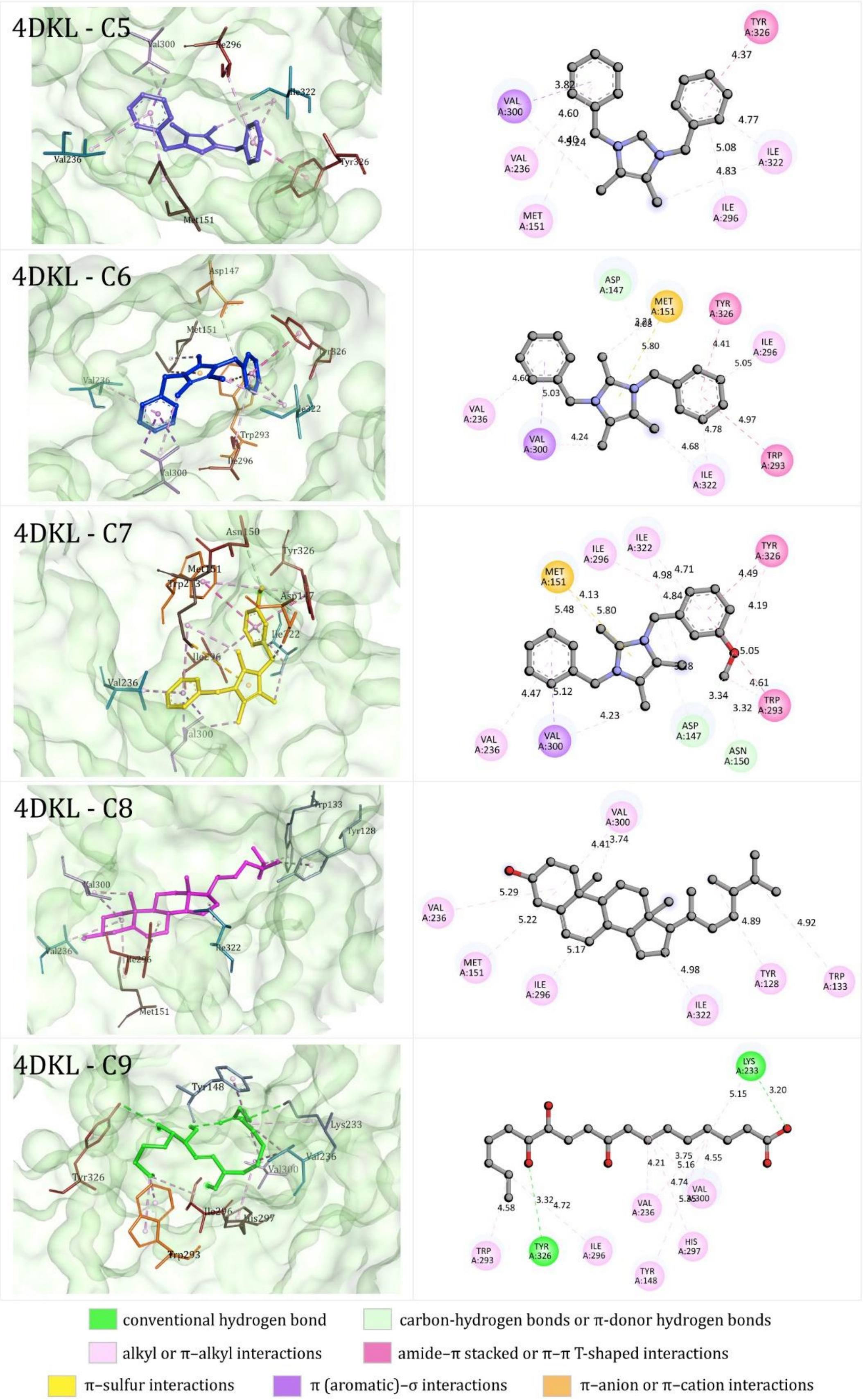
4DKL - C5
4DKL - C6
4DKL - C7
4DKL - C8
4DKL - C9
conventional hydrogen bond
carbon-hydrogen bonds or π-donor hydrogen bonds
alkyl or π–alkyl interactions
amide–π stacked or π–π T-shaped interactions
π–sulfur interactions
π (aromatic)–σ interactions
π–anion or π–cation interactions

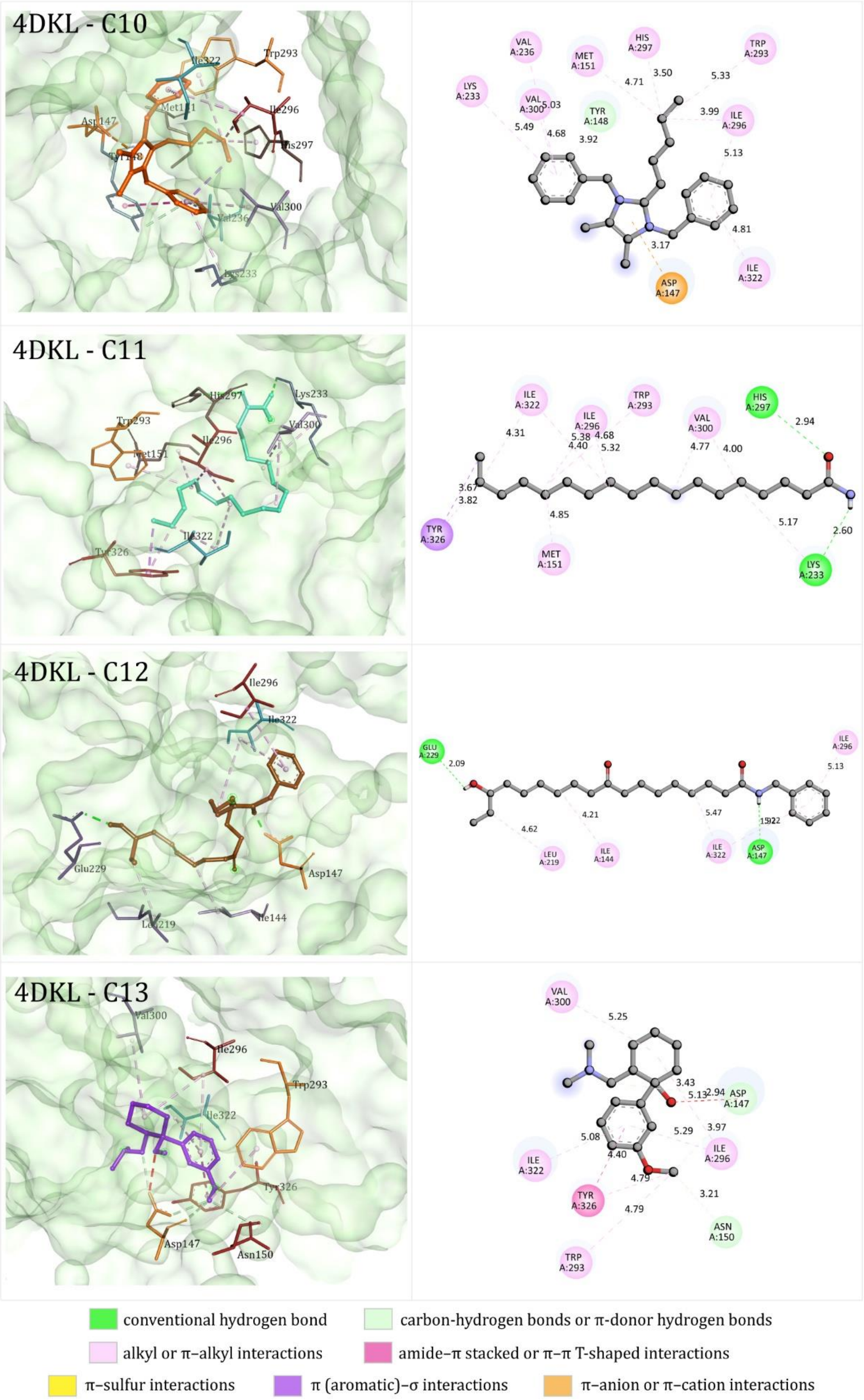
4DKL - C10
4DKL - C11
4DKL - C12
4DKL - C13
conventional hydrogen bond
carbon-hydrogen bonds or π-donor hydrogen bonds
alkyl or π–alkyl interactions
amide–π stacked or π–π T-shaped interactions
π–sulfur interactions
π (aromatic)–σ interactions
π–anion or π–cation interactions

Figure S6. 2D representation of the interactions between compounds **1**-**12** and residues of the fatty acid amide hydrolase (FAAH, PDB ID: 2VYA). Hydrogen atoms have been omitted in some cases for clarity.

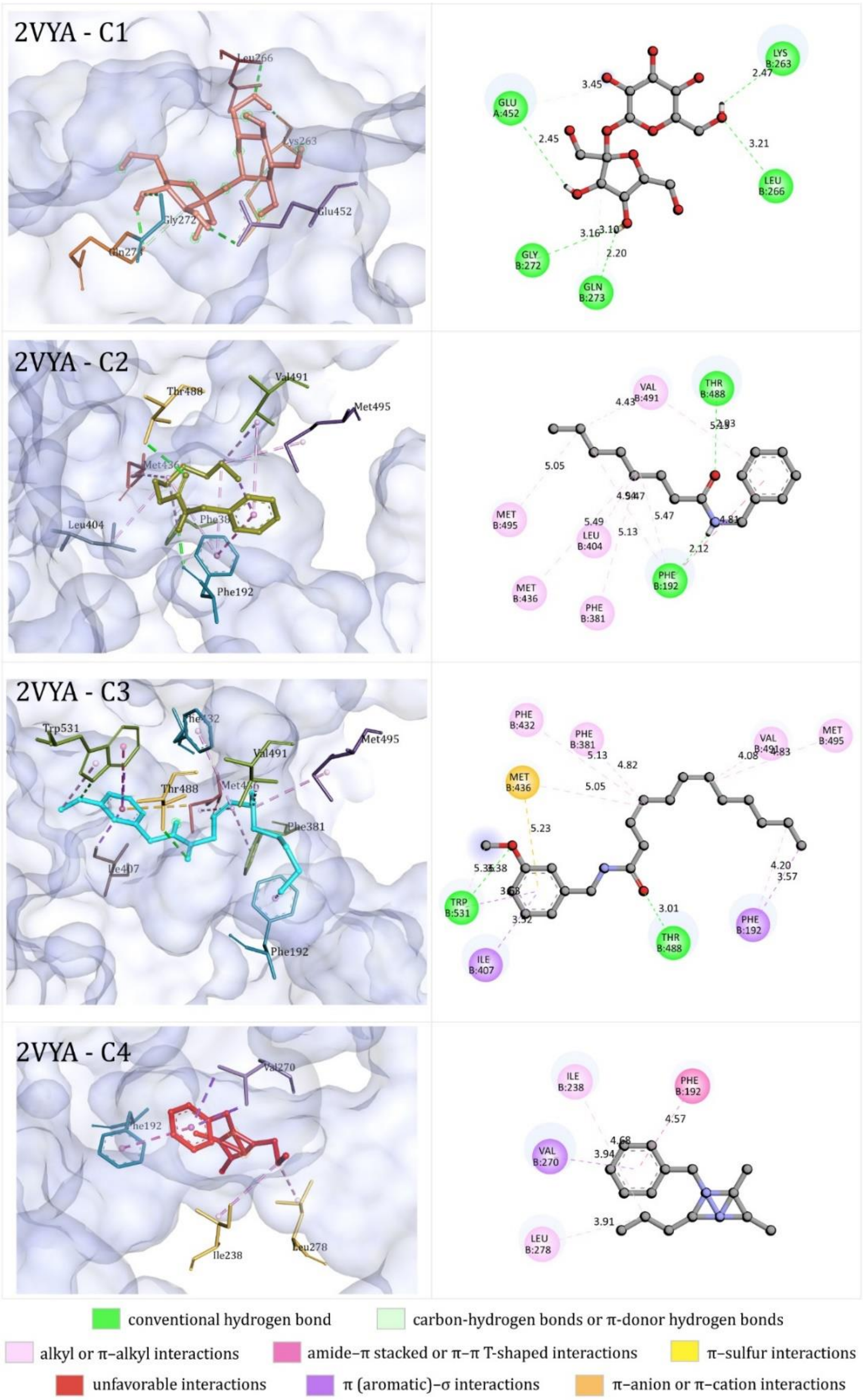

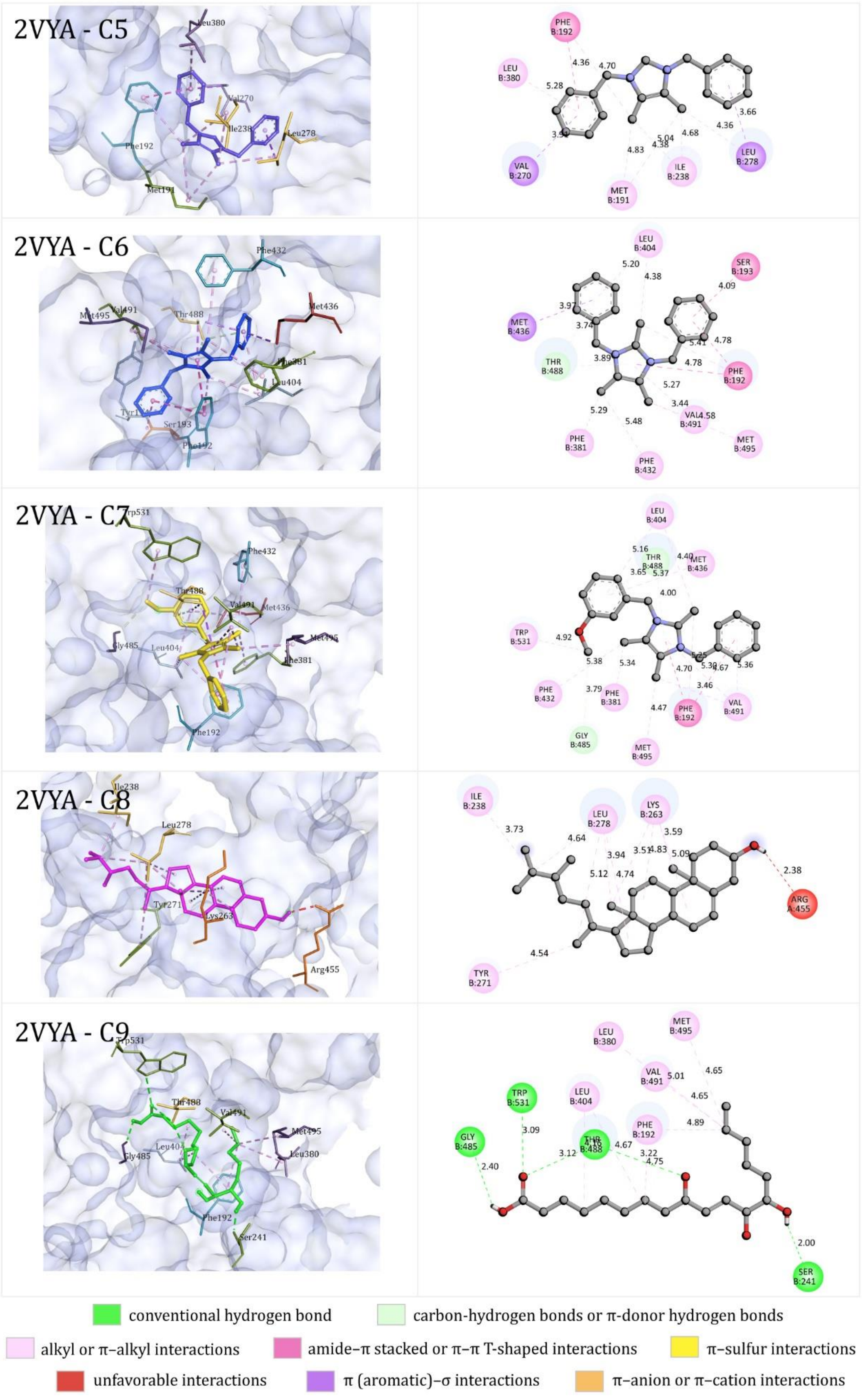
2VYA - C5
2VYA - C6
2VYA - C7
2VYA - C8
2VYA - C9
conventional hydrogen bond
carbon-hydrogen bonds or π-donor hydrogen bonds
alkyl or π–alkyl interactions
amide–π stacked or π–π T-shaped interactions
π–sulfur interactions
unfavorable interactions
π (aromatic)–σ interactions
π–anion or π–cation interactions

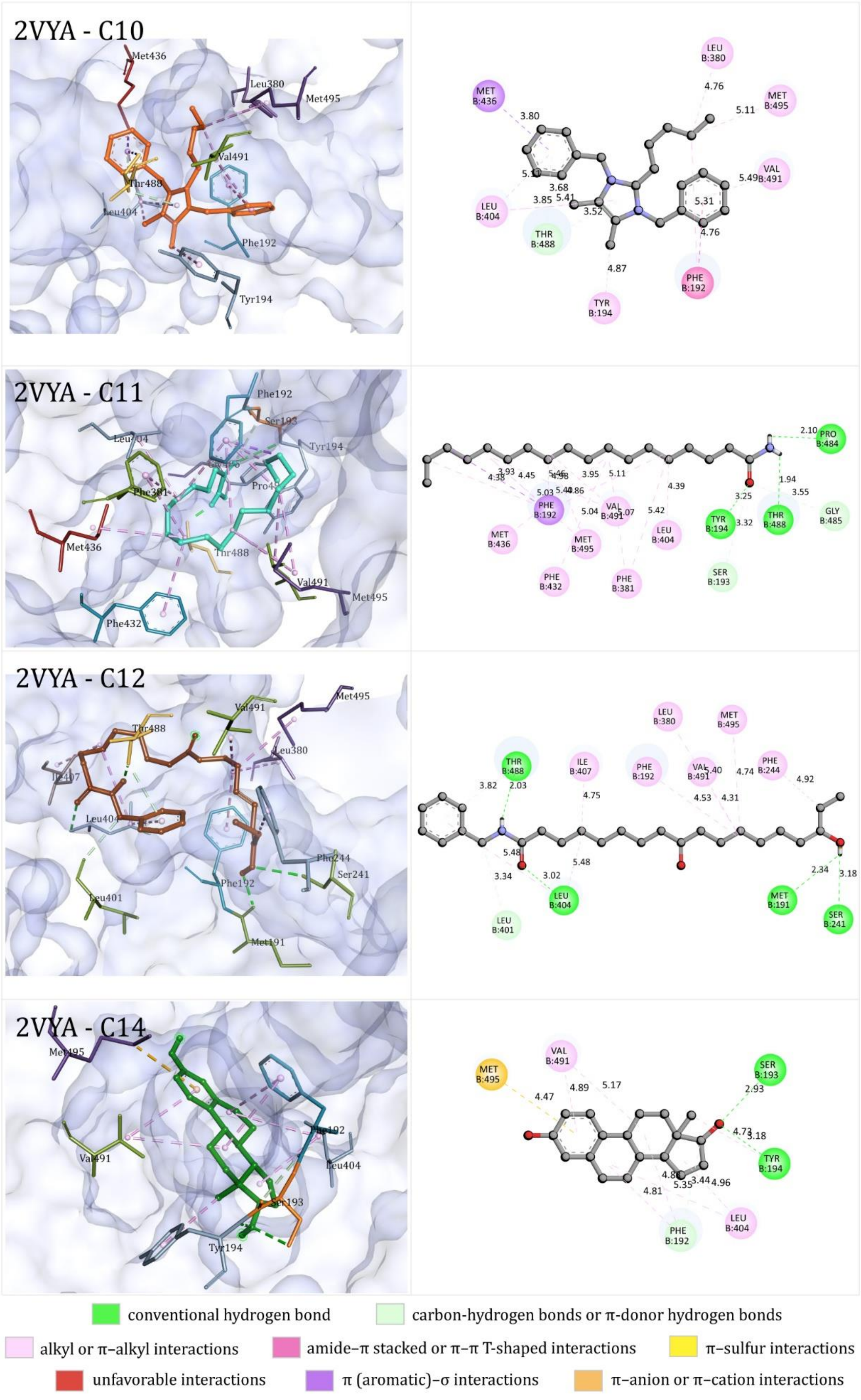
2VYA - C10
2VYA - C11
2VYA - C12
2VYA - C14
conventional hydrogen bond
carbon-hydrogen bonds or π-donor hydrogen bonds
alkyl or π–alkyl interactions
amide–π stacked or π–π T-shaped interactions
π–sulfur interactions
unfavorable interactions
π (aromatic)–σ interactions
π–anion or π–cation interactions

General note:

Proteins are shown in gray, crystallographic ligand poses in blue, and re-docked ligand poses in color (as indicated in each figure). The root-mean-square deviation (RMSD) between crystallographic and re-docked poses was below 2.0 Å, validating the docking protocol.

Figure S7. Superimposition of the crystallographic and re-docked ligand poses in the active site of NADPH oxidase (PDB ID: 2CDU). Re-docked ligand shown in pink.

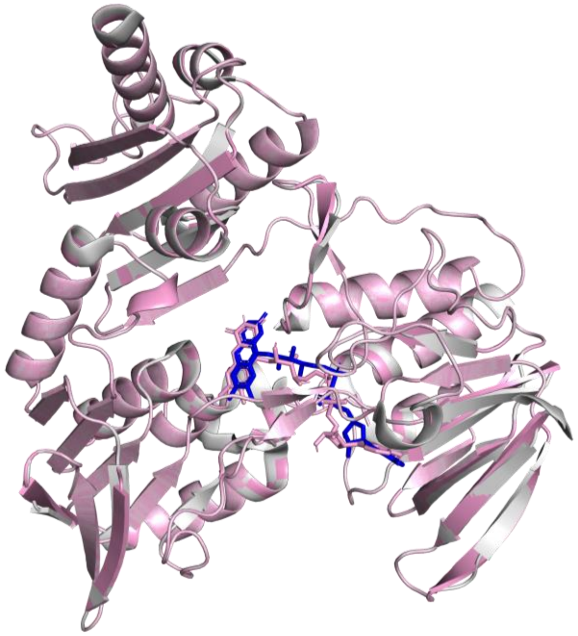

Protein = gray and ligand = blue → Experimental ligand conformation

Protein 2CDU and ligand = pink → Predicted docked pose obtained by re-docking

Figure S8. Superimposition of the crystallographic and re-docked ligand poses in the active site of xanthine oxidase (PDB ID: 3NRZ). Re-docked ligand shown in light blue.

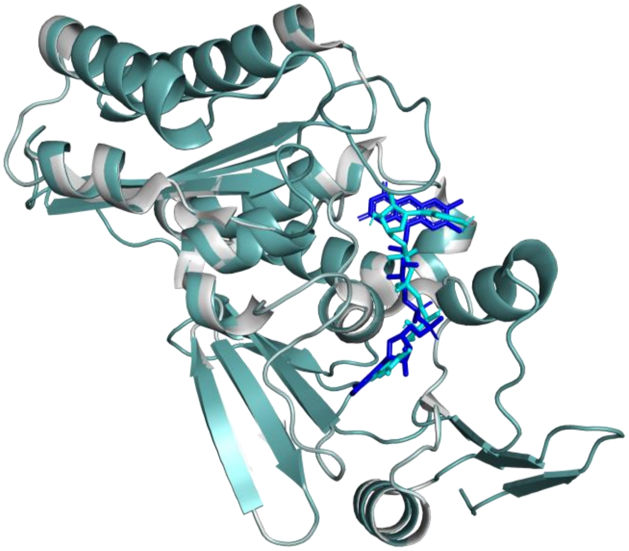

Protein = gray and ligand = blue → Experimental ligand conformation

Protein 3NRZ and ligand = light blue → Predicted docked pose obtained by re-docking

Figure S9. Superimposition of the crystallographic and re-docked ligand poses in the active site of superoxide dismutase (PDB ID: 4MCM). Re-docked ligand shown in yellow.

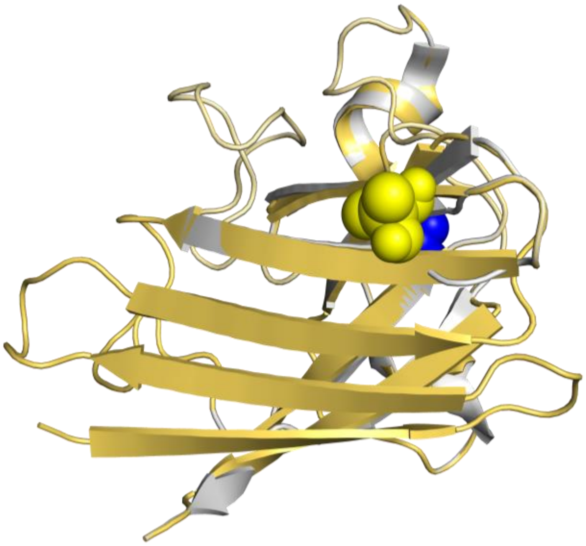

Protein = gray and ligand = blue → Experimental ligand conformation

Protein 4MCM and ligand = yellow → Predicted docked pose obtained by re-docking

Figure S10. Superimposition of the crystallographic and re-docked ligand poses in the active site of the µ-opioid receptor (PDB ID: 4DKL). Re-docked ligand shown in green.

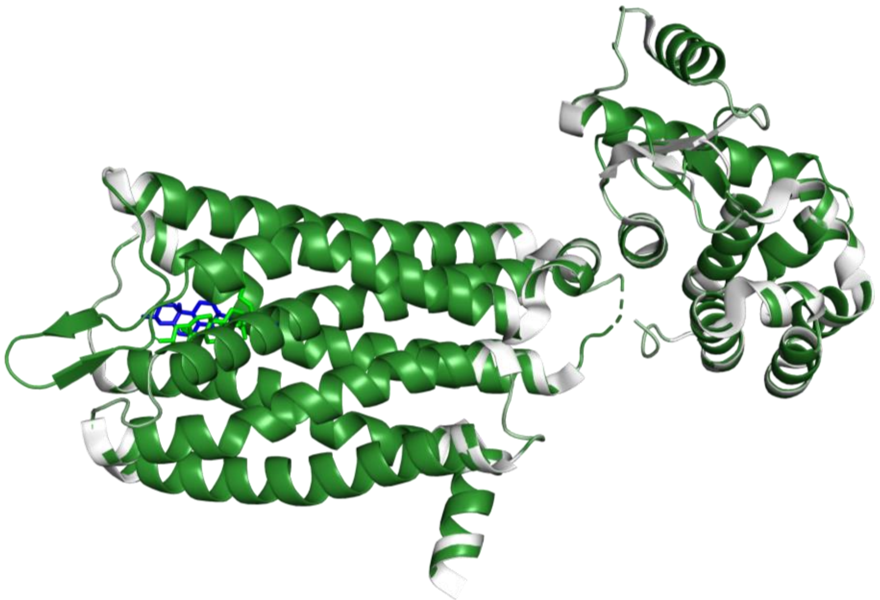

Protein = gray and ligand = blue → Experimental ligand conformation

Protein 4DKL and ligand = green → Predicted docked pose obtained by re-docking

Figure S11. Superimposition of the crystallographic and re-docked ligand poses in the active site of fatty acid amide hydrolase (FAAH) (PDB ID: 2VYA). Re-docked ligand shown in purple.

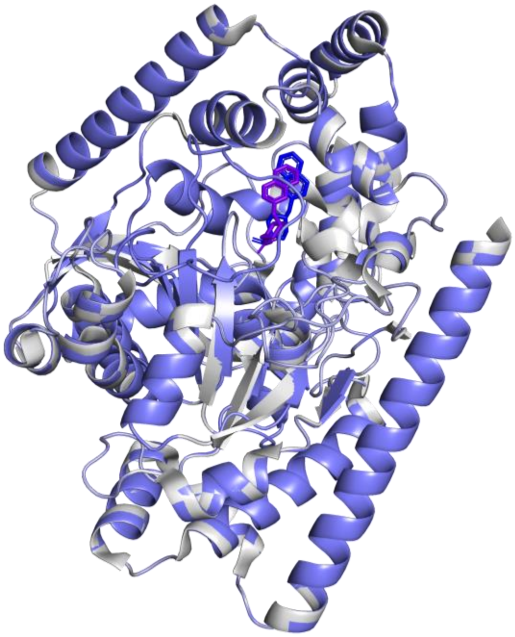

Protein = gray and ligand = blue → Experimental ligand conformation

Protein 2VYA and ligand = purple → Predicted docked pose obtained by re-docking
